# A Primer to (Cross-Cultural) Multi-Group Invariance Testing Possibilities in R

**DOI:** 10.3389/fpsyg.2019.01507

**Published:** 2019-07-18

**Authors:** Ronald Fischer, Johannes A. Karl

**Affiliations:** ^1^School of Psychology and Center for Applied Cross-Cultural Psychology, Victoria, Wellington, New Zealand; ^2^Instituto D’Or de Pesquisa e Ensino, São Paulo, Brazil

**Keywords:** invariance, culture, procrustean analyses, confirmatory factor analysis – CFA, DIF (differential item functioning), R, ESEM, alignment

## Abstract

Psychology has become less WEIRD in recent years, marking progress toward becoming a truly global psychology. However, this increase in cultural diversity is not matched by greater attention to cultural biases in research. A significant challenge in culture-comparative research in psychology is that any comparisons are open to possible item bias and non-invariance. Unfortunately, many psychologists are not aware of problems and their implications, and do not know how to best test for invariance in their data. We provide a general introduction to invariance testing and a tutorial of three major classes of techniques that can be easily implemented in the free software and statistical language R. Specifically, we describe (1) confirmatory and multi-group confirmatory factor analysis, with extension to exploratory structural equation modeling, and multi-group alignment; (2) iterative hybrid logistic regression as well as (3) exploratory factor analysis and principal component analysis with Procrustes rotation. We pay specific attention to effect size measures of item biases and differential item function. Code in R is provided in the main text and online (see https://osf.io/agr5e/), and more extended code and a general introduction to R are available in the Supplementary Materials.

## Introduction

We live in an ever increasingly connected world and today it is easier than ever before to administer surveys and interviews to diverse populations around the world. This ease of data gathering with instruments often developed and validated in a single region of the world is matched by the problem that it is often difficult to interpret any emerging differences (for a discussion see: Chen, 2008; Fischer and Poortinga, 2018). For example, if a researcher is interested in measuring depression or well-being, it is important to determine whether the instrument scores can be compared across cultural groups. Is one group experiencing greater depression or psychological distress compared to another group? Hence, before we can interpret results in theoretical or substantive terms, we need to rule out methodological and measurement explanations. Fortunately, the methods have advanced significantly over the last couple of years, with both relatively simple and increasingly complex procedures being available to researchers (Vandenberg and Lance, 2000; Boer et al., 2018). Some of the more advanced methods are implemented in proprietary software, which may not be available to students and researchers, especially in lower income societies. There are excellent free and online resources available, most notable using the programming language R (R Core Team, 2018). Unfortunately, many researchers are not aware of the interpretational problems in cross-cultural comparative research and fail to adequately test for measurement invariance (see Boer et al., 2018). Our tutorial aims to demonstrate how three different and powerful classes of analytical techniques can be implemented in a free and easy to use statistical environment available to student and staff alike which requires little computer literacy skills. We provide the code and example data in the Supplementary Materials as well as online^1^. We strongly encourage readers to download the data and follow the code to gain some experience with these analyses.

We aim to provide a basic introduction that allows novices to understand and run these techniques. The three most common approaches are exploratory and confirmatory methods within the classic test theory paradigm as well as item response theory approaches. We also include recent extension such as exploratory structural equation modeling (ESEM) and multi-group alignment. Although these approaches often differ at the philosophical and theoretical level, at the computational level and in their practical implementation, they are typically converging (Fontaine, 2005). We provide a basic introduction and discuss them together here. We encourage readers interested in more technical discussions and their conceptual and computational distinctions to consult more technical overviews and extensions (e.g., Long, 1983a,b; Hambleton and Jones, 1993; Meredith, 1993; Fontaine, 2005; Borsboom, 2006; Tabachnick and Fidell, 2007; Boer et al., 2018).

Throughout the tutorial, we use a two-group comparison. Unfortunately, results from two sample comparisons are open to a host of alternative interpretations, even if method issues can be ruled out. Therefore, we strongly encourage researchers to include more than two samples in their research design. Multiple-sample can pose some additional analytical choices for researchers (especially for the EFA component) and we discuss easily available options for expanding the analyses to more than two samples. In the final section, we directly compare the different methods and their relative advantages and disadvantages.

## The Basic Principle of Measurement Invariance Testing

**FIGURE 1:**
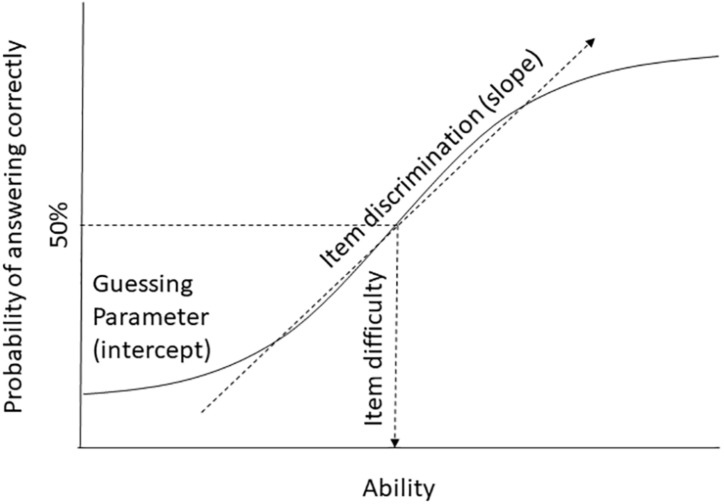
Schematic display of item difficulty, item discrimination, and guessing parameters in a single group.

With invariance testing, researchers are trying to assess whether an instrument has the same measurement properties in two or more populations. We need to distinguish a number of different properties of measurement instruments. In order to provide a common terminology, we use the item response theory approach (we will be ignoring the person parameters) and note equivalent parameters in classic test theory terms, where necessary. Because in psychology we often do not have access to objective indicators, our best estimate about the psychological expression of interest when evaluating a test is the overall score on a test. This overall score is taken as an estimate of the underlying ability parameter of the person or the level of latent variable (the psychological trait we would like to measure). Invariance testing of instruments focuses on the relationship between each individual item and the overall score of the instrument. It is important to highlight that cross-cultural researchers use different types of data for invariance testing and that the interpretation of the overall score differs depending on the type of test being examined. For example, an intelligence test will capture the extent to which individuals answer questions correctly, which then leads to clear interpretations of the parameters in terms of item difficulty and item discrimination. For researchers using rating scales, these same parameters are often interpreted in terms of factor loadings (how well an item relates to a presumed underlying factor) and intercepts (is there some guessing or response bias involved, that is not related to the latent variable). The interpretation therefore differs somewhat, but the statistical properties are similar. For example, if an individual has a higher score on the underlying ability as either a true ability or a preference or trait, then she should report a higher mean (the person is more likely to answer an item ‘correctly’). When dissecting the relationship between an item and the overall score, there are three main parameters: (1) the item difficulty or item location, (2) the discrimination or slope parameter, and (3) a parameter for pseudo-guessing, chance or the intercept (see Figure 1). The item difficulty describes how easy or difficult an item is, in other words, the amount of a latent trait that is needed for an individual to endorse an item with a 50% probability (for rating scales) or answer it correctly (for ability tests). Item discrimination or the slope describes how well an item discriminates between individuals (both for ability tests and rating scales). In factor analytic terms it can also be thought of as the item loading – how strongly the item is related to the latent variable. The guessing parameter refers to the point where individuals with a low level of ability (for ability tests) or expression of a psychological trait (for rating scales) may still able to guess the correct answer (on a test) or responds with a higher score than would be indicated by their latent trait score. In factor analytic terms, this is conceptually equivalent to the intercept. More parameters can be estimated and tested (in particular within a multivariate latent variable framework), but these three parameters have been identified as most important for establishing cross-cultural measurement invariance (e.g., Meredith, 1993; Vandenberg and Lance, 2000; Fontaine, 2005). Of these three parameters, item discrimination and intercepts are the most central and have been widely discussed in terms of how they produce differential item functioning (DIF) across groups.

## Levels of Measurement Invariance and Differential Item Bias

**FIGURE 2:**
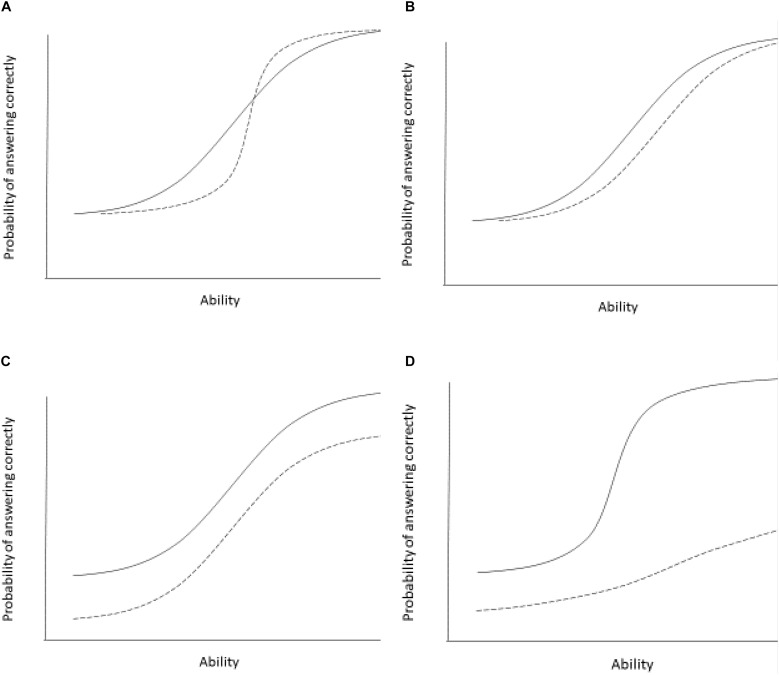
Examples of differential item functioning in two groups. The panels show differential item functioning curves for two groups (group 1 indicated by solid line, group 2 indicated by a broken line). Panel **(A)** shows two groups differing in item discrimination (slope differences). The item differentiates individuals less well in group 1. This is an example of non-uniform item bias. Panel **(B)** shows two groups with different item difficulty. The item is easier (individuals with lower ability are able to correctly answer the item with 50% probability) for the group 1 and more difficult for group 2. Individuals in group 2 need higher ability to answer the items correctly with a 50% probability. This is an example of uniform item bias. Panel **(C)** shows differential guessing or intercept parameters. Group 1 has a higher chance of guessing the item correctly compared to group 2. Scores for group 1 on this item are consistently higher than for group 2, independent of the individual’s underlying ability or trait level. This is an example of uniform item bias. Panel **(D)** shows two groups differing in all three parameters. Group 1 has a higher guessing parameter, the item is easier overall, but also discriminates individuals better at moderate levels of ability compared to group 2. This is an example of both uniform and non-uniform item bias.

In cross-cultural comparisons, it is important to identify whether these parameters are equivalent across populations, to rule out the possibility that individuals with the same underlying ability have a different probability to give a certain response to a specific item depending on the group that they belong to (see Figure 2).

There are at least three different levels of invariance or equivalence that are often differentiated in the literature (see Meredith, 1993; van de Vijver and Leung, 1997; Vandenberg and Lance, 2000; Fontaine, 2005; Milfont and Fischer, 2010). The first issue is whether the same items can be used to measure the theoretical variable in each group. For example, is the item “I feel blue” a good indicator of depression?^2^ If the answer is yes, we are dealing with configural invariance. The loadings (the extent to which each item taps into the underlying construct of depression) are all in the same direction in the different groups (this is why this sometimes called form invariance), nevertheless, the specific factor loadings or item discrimination parameters may still differ across samples.

If the item discrimination or factor loadings are identical across the samples, then we are dealing with metric invariance. The item discriminates similarly well between individuals with the same underlying trait. Equally, the item is related to the same extent to the underlying latent variable in all samples. This implies that an increase in a survey response to an item (e.g., answering with a 3 on 1–7 Likert scale instead of a 2) is associated with the same increase in depression (the latent variable that is thought to cause the responses to the survey item) in all groups sampled. If this condition is met for all items and all groups, we can compare correlations and patterns of means (e.g., profiles) across cultural samples, but we cannot make claims about any latent underlying construct differences (see Fontaine, 2005).

See Figures 2B,C for an example where an increase in the underlying ability of trait is associated with equal changes in responses to an individual item, but there are still other parameters that differ between samples.

If we want to compare instrument scores across groups and make inferences about the underlying trait or ability levels, we need to also at least constrain guessing or intercept parameters (and also item difficulty in IRT). Metric invariance only means that the slopes between items and latent variables are identical, but the items may still be easier or difficult overall or individuals might be able to guess answers. Therefore, we have to constrain intercepts to be equal. If this condition is met, we have scalar or full score invariance. The advantage of full score equivalence is that we can directly compare means and interpret any differences in terms of the assumed underlying psychological construct.

These levels of invariance are challenged by two major item biases. Uniform item bias describes a situation where the item equally well discriminates between individuals with the same underlying true ability. In this case the curves are parallel and the items do not differ in discrimination (slopes). People of one group have an unfair advantage over the other group, but the relative order of individuals within each group is preserved (see Figures 2B,C). Non-uniform item bias occurs when the order of individuals along the true underlying trait is not reflected in the item responses (see Figures 2A,D). The item responses differ across groups and true levels of the underlying ability. The most important parameter here is item discrimination, but other parameters may also change. Together, these item biases are often examined in the context of DIF.

The methods discussed below differ in the extent to which they allow researchers to identify item bias and invariance in these parameters. Exploratory factor analysis (EFA) with Procrustes rotation is the least rigorous method, because it only allows an overall investigation of the similarity of factor loadings, but it does not typically allow analysis at the item level. Multi-group confirmatory factor analysis (CFA) and DIF analysis with logistic regression allow an estimation of both the similarity in factor loadings and intercepts/guessing parameters. We briefly describe the theoretical frameworks, crucial analysis steps and how to interpret the outputs in a two-group comparison. We then compare the relative advantages and disadvantages of each method and their sensitivity to pick up biases and violations of cross-cultural invariance.

### What to Do if Invariance Is Rejected?

All the techniques that we describe below rely on some form of fit statistic – how much does the observed data deviate from the assumption that the statistical parameters are equal across groups? The different techniques use different parameters and ways to test this misfit, but essentially it always comes down to an estimation of the deviation from an assumed equality of parameters. Individual items or parameters are flagged for misfit. The most common immediate strategy is to conduct exploratory analyses to identify (a) the origin of the misfit or DIF and to then (b) examine whether excluding specific items, specific factors or specific samples may result in improved invariance indicators. For example, it might be that one item shows some translation problems in one sample and it is possible to exclude this item or to run so-called partial invariance models (see below). Or there might be problems with a specific factor (e.g., translation problems, conceptual issues with the psychological meaning of factor content – often called cultural construct bias). It might be possible to remove the factor from the analyses and proceed with the remaining items and factors. Or it may also happen that one sample is found to be quite different (e.g., different demographics or other features that distinguish the sample from the other samples including differences in reading ability, education, economic opportunities). In this case, it is possible to exclude the individual sample and proceed with the remaining cultural samples. The important point here is that the researcher needs to carefully analyze the problem and decide whether it is a problem with an individual item, scale or sample, or whether it points to some significant cultural biases at the conceptual level.

We would like to emphasize that it is perfectly justified to conduct an invariance analysis and to conclude that it is not meaningful to compare results across groups. In fact, we wish more researchers would take this stance and call out test results that should not be compared across ethnic or cultural groups. For example, if the factor structures of an instrument are not comparable across two or more groups, a comparison of means and correlations are invalid. There is no clear interpretation of any mean differences if there is no common structure. Hence, invariance analysis can be a powerful tool for applied psychologists to counter discrimination and bias as well as cultural psychologists interested in demonstrating cultural relativism. Unfortunately, too often the insights from invariance analyses are ignored and researchers proceed with cross-cultural comparisons, which are then inherently meaningless (see Boer et al., 2018).

## Confirmatory Factor Analysis

**FIGURE 3:**
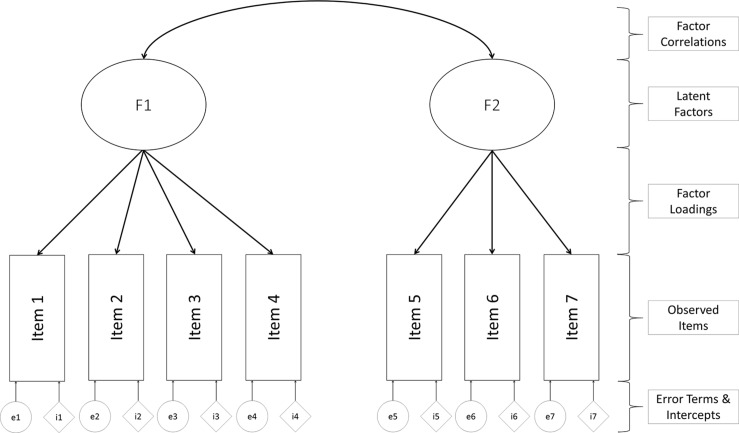
Example of confirmatory factor analysis model.

Confirmatory factor analysis is probably the most widespread measurement model approach in psychology. Most constructs in psychological research cannot be directly observed but need to be inferred from several observed indicators (Horn, 1965; Gorsuch, 1993). These indicators can be recorded behaviors or responses to Likert type scales: returning to our example of depression, we may infer levels of an underlying depression variable through observations of sleeping problems, changes in mood, or weight gain. The general advantage and appeal of CFA is that it explicitly tests the theoretical structure that a researcher has about the instrument. CFA using a theory-driven approach for modeling the covariance between items, meaning it is a measurement model that treats items as indicators of a theoretically assumed underlying latent constructs (e.g., Long, 1983a; Bollen, 1989). The researcher needs to decide *a priori* which items are expected to load (are indicators of the latent variable) on which latent variable. Typically, researchers are interested in the simple structure, in which each item is expected to load on only one latent factor. Figure 3 shows the main parts of a CFA model. Observed indicators (e.g., item responses to a survey) are represented by squares, whereas estimated parameters are symbolized by ovals or circles. Each item in our example is allowed to load on one latent variable. The resulting factor loadings represent the relationship of the observed indicator to each of the extracted latent factors. The strength of the loadings can range from 0 (no relationship) to either −1 or 1 (identical); if the latent variables are standardized, in unstandardized situations the loadings are dependent on the measurement scale. In our example, the first four items only load on factor 1, whereas the last three items only load on factor 2. In multi-group analyses, we also estimate the item intercept (which is conceptually similar to the pseudo guessing parameter discussed above).

For technical (identification) purposes, one of the factor loadings is typically set to 1 to provide identification and a meaningful scale. It also important to have at least three items per latent factor (although this rule can be relaxed, see Bollen, 1989). CFA is demanding in terms of data quality, assuming at least interval data that is multivariate normally distributed, an assumption that is unfortunately often violated. Some procedures have been developed to correct for a violation of multivariate normality (see for example, Satorra and Bentler, 1988), which are implemented and can be requested in the R package that we describe below.

Confirmatory factor analysis is confirmatory: the theoretically proposed structure of implied covariances among items is statistically tested and compared to the observed covariances based on the sample specific item responses. One of the most important questions is how to evaluate whether the model fits the data. Various different fit indices are available. The deviation of the theoretically predicted to the empirically observed covariances is captured by the chi-square statistic. This is the oldest and probably most important diagnostic tool for deciding whether the theoretical prediction was plausible or not. The smaller the chi-square value, the less the theoretical model deviates from the observed sample covariance matrix. The exact fit of the theory to the data can be evaluated with a significance test, therefore this is often called an exact fit test (see Barrett, 2007). Ideally, we want a non-significant chi-square value. Unfortunately, there are both conceptual and statistical drawbacks for the chi-square. First, any theoretical model is only an approximation of reality, therefore any chi-square is *a priori* known to be incorrect and bound to fail because reality is more complex than implied in simple models (Browne and Cudeck, 1992). Statistically, the test is sample size dependent. Any model will be rejected with a sufficiently large sample size (Bentler and Bonett, 1980; Bollen, 1989; for an example of cross-cultural study demonstrating this dependence, see Fischer et al., 2011).

To overcome these problems, a number of alternative fit measures have been proposed (even though most of them still are derived from the qui-square statistic). Here, we focus on the most commonly reported fit statistics (Hu and Bentler, 1999), which can be differentiated into (a) incremental or comparative and (b) lack-of-fit indices. Incremental or comparative fit models compare the fit of the theoretical model against an alternative model. This is (typically) an independence model in which no relationships between variables are expected. Higher values are indicating better fit with values above 0.95 indicating good fit (Hu and Bentler, 1998). The Tucker–Lewis Index (TLI) or non-normed fit index (NNFI) and the comparative fit index (CFI; Bentler, 1990) are the most commonly reported and more robust indicators (Hu and Bentler, 1999). Lack of fit indices in contrast indicate better fit, if the value is lower. The standardized root mean square residual (SRMR; Bollen, 1989) compares the discrepancy between the observed correlation matrix and the implied theoretical matrix. Smaller values indicate that there is less deviation. Hu and Bentler (1998) suggested that values less than 0.08 are acceptable. The root mean square error of approximation (RMSEA; Browne and Cudeck, 1992) uses a similar logic, but also takes into account model complexity and rewards more parsimonious models. Historically, values ranging between 0.06 and 0.08 were deemed acceptable, but simulations by Hu and Bentler (1998, 1999) suggested that a cut-off of 0.06 might be more appropriate.

However, it is important to note that the selection of fit indices and their cutoff criteria are contentious. Marsh et al.’s (2004) study warned researchers against blindly adopting cutoff values suggested by specific simulations such as the famous Hu and Bentler (1998, 1999) study. One specific issue is that models with higher factor loadings (indicating more reliable models) might be penalized by these fit indicators (Kang et al., 2016; McNeish et al., 2018), which creates a paradoxical situation in that theoretically better and more reliable models are showing worse fit. They suggested to also examine other fit indices such as McDonald’s Non-Centrality Index (NCI, McDonald, 1989). We urge researchers to take a cautious approach and to evaluate model fit as well as examining the overall factor loadings and residuals when determining model fit. If your model is fitting well, but has poor factor loadings and shows large residuals, it is probably not the best model. A good strategy is to compare a number of theoretically plausible models and then select the model that makes most theoretical sense and has the best fit (MacCallum and Austin, 2000; Marsh et al., 2004).

Often, researcher would first test the model separately in each cultural group. This can provide valuable insights into the structure in each group. However, the individual analyses in each sample do not provide information about whether the structure is identical or not across groups. For this, we need to conduct a multi-group analysis. This is the real strength of CFA, because we can constrain relevant parameters across groups and test whether the fit becomes increasingly worse. If there is overall misfit, it then becomes possible to test whether individual items or groups cause misfit. Therefore, multi-group CFA provides information at both the scale and item level, making it a powerful tool for cross-cultural researchers.

To proceed with the examination of invariance, a number of parameters can be constrained across groups or samples in a hierarchical fashion which allow a test of the invariance levels that we described at the beginning of this article. The first step is form invariance (Meredith, 1993; Cheung and Rensvold, 2000) or configural invariance (Byrne et al., 1989). All items are expected to load on the same latent factor. The second level is factorial invariance (Cheung and Rensvold, 2000) or metric invariance (Byrne et al., 1989), in which the factor loadings are forced to be equal across groups. This tests whether there is non-uniform item bias (see above). The third level that is necessary to test is scalar invariance (Vandenberg and Lance, 2000) or intercept invariance (Cheung and Rensvold, 2000), which constrains the item intercepts to be equal across groups. It tests whether there is uniform item bias present in an item. It is desirable to obtain scalar invariance because then means can be directly compared across groups. Unfortunately, few cross-cultural studies do test this level of invariance (Boer et al., 2018).

At each step, researchers have to decide whether their more constrained model still fits the data or not. In addition to the fit indices that we have discussed above, it is common to examine change in fit statistics. The traditional change statistic is the chi-square difference test, in which the chi-square of the more restricted model is compared to the chi-square of the more lenient model. A significant chi-square difference indicates that model fit is significantly worse in the more restricted model (Anderson and Gerbing, 1988). However, as before, the chi-square is sample size dependent and therefore, other fit indices have been introduced. Little (1997) was the first to suggest that differences in the NNFI/TLI and CFI are informative. Similarly, it is possible to examine changes in RMSEA (Little et al., 2007). For these change in fit indices, current standards are to accept models that show differences equal to or less than 0.01. Some authors also suggested examining other fit indices, including ΔMcDonald’s NCI (see Kang et al., 2016). All these fit indices are judged in relation to deterioration in fit between more and less restricted models, with cut-offs based on either experience or simulations. Unfortunately, there is no universal agreement on acceptable standards (see Chen, 2007; Milfont and Fischer, 2010; Putnick and Bornstein, 2016). For example, Rutkowski and Svetina (2014) ran simulation models focusing specifically on conditions where researchers have more than 10 samples in their measurement invariance analysis and suggested that in these multi-group conditions criteria for metric invariance tests could be relaxed to 0.02, but that the criteria for judging scalar invariance should remain at traditional cut-offs of less than 0.01.

What do you need to do if factorial invariance is rejected at any of these steps? First, it is advisable to investigate the models in each group separately and to also check modification indices and residuals from the constrained model. Modification indices provide information of how much the χ^2^ would change if the parameter was freed up. There are no statistical guidelines of how big a change has to be in order to be considered meaningful. Theoretical considerations of these modification indices are again important: There might be both meaningful theoretical (conceptual differences in item meaning) or methodological reasons (item bias such as translation issues, culture specificity of item content, etc.) why either factor loadings or intercepts are different across groups. The appropriate course of action depends on the assumed reasons for misfit. For example, a researcher may decide to remove biased items (if there are only few items and if this does not threaten the validity of the overall scale). Alternatively, it is possible to use partial invariance, in which the constrains on specific items are relaxed (Byrne et al., 1989, see below).

### How to Run a Multi-Group CFA in R

We describe the steps using the lavaan (Rosseel, 2012) and semTools (semTools Contributors, 2016) packages, which need to be loaded (see Supplementary Materials). For illustration purposes, we use data from Fischer et al. (2018) in which they asked employees in a number of countries, including Brazil and New Zealand (total *N* = 2,090, we only included a subset of the larger data set here), to report whether they typically help other employees (helping behavior, seven items) and whether they make suggestions to improve work conditions and products (voice behavior, five items). Individuals responded to these items on a 1–7 Likert-type scale.

#### Running the CFA

The first CFA relevant step after reading in the data and specifying missing data (see Supplementary Materials) is to specify the theoretical model. We need to create an object that contains the relevant information, e.g., what item loads on what factor and whether factors and/or item errors are correlated. The way this is done is through regression-like equations. Factor loadings in R are indicated by =∼ and covariances (between factors or error terms for items) are indicated by ∼∼. The model is specified similar to writing regression equations.

In our case, the model is:

cfa_model<− ‘

help =∼help1 + help2 + help3 + help4 + help5 + help6 + help7

voice =∼voice1 + voice2 + voice3 + voice4 + voice5’

We have seven items that measure helping behavior and five items that measure voice behaviors. Now, we need to run the model and test whether the theoretical model fits to our data. The basic command is:

fit_cfa <− cfa(cfa_model, data = example)

#### Running a Multi-Group CFA

This creates an object that has the statistical results. The current command does not specify separate CFAs in the individual groups, but tests the model in the total sample. To separate the models by group, we need to specify the group (important note: in lavaan, we will not get the separate fit indices per group, but only an overall fit index for all groups combined; if you want to run separate CFAs in each group, it is useful to subset the data first, see the Supplementary Materials for data handling):

fit_cfa_country <− cfa(cfa_model, data = example,

group = “country”)

To get the statistical output and relevant fit indices, we can now call the object that we just created in the summary() function:

summary(fit_cfa_country, fit.measures = TRUE,

standardized = TRUE, rsquare = TRUE)

The fit.measures argument requests the commonly described fit indices that we described above. The standardized command provides a standardized solution for the loadings and variances that is more easily interpreted. In our case, the fit is mixed overall: χ^2^(106) = 928.06, *p* < 0.001, CFI = 0.94, TLI = 0.93, RMSEA = 0.086, SRMR = 0.041. For illustration purposes, we continue with this model, but caution that it is probably not demonstrating sufficient fit to be interpretable.

#### Invariance Testing – Omnibus Test

To run the invariance analysis, we have two major options. One is to use a single command from the semTools package which runs the nested analyses in a single run:^3^

measurementInvariance (model = cfa_model, data =

example, group = “country”)

We specify the theoretical model to test, our data file and the grouping variable (country). In the output, Model 1 is the most lenient model, no constraints are imposed on the model and separate CFA’s are estimated in each group. The fit indices mirror those reported above. Constraining the loadings to be equal, the difference in *χ^2^* between Model 1 and 2 is not significant: Δχ^2^(df = 10) = 16.20, *p* = 0.09, and the change in both CFI (0.00) and RMSEA (0.003) are negligible. Since *χ^2^* is sensitive to sample size, the CFI and RMSEA parameters might be preferable in this case (see Milfont and Fischer, 2010; Cheung and Lau, 2012; Putnick and Bornstein, 2016). When further constraining the intercepts to be equal, we have a significant *χ^2^* difference again: Δχ^2^(df = 10) = 137.03, *p* < 0.001. The difference in CFI (0.009) and RMSEA (0.003) are also below commonly accepted thresholds, therefore, we could accept our more restricted model. However, as we discussed above, the overall fit of the baseline model was not very good and some of the fit indices have conceptual problems. In the ccpsyc package, we included a number of additional fit indices that have been argued to be more robust (see for example, Kang et al., 2016). Briefly, to load the ccpsyc package (the devtools package is required for installation), call this command:

devtools::install_github(”Jo-Karl/ccpsyc”)

library(ccpsyc)

The function via the ccpsyc package is called equival and we need to specify the CFA model that we want to use, then the relevant data file (dat = example) and the relevant grouping variable (group = “country”). For this function, the group variable needs to be a factor (e.g., the country variable is not a numerical variable). It is important to note that the *equival* function fits all models using a robust MLM estimator rather then an ML estimator.

An example of the function is:

equival(cfa_model, dat = example, group = “country”)

In our previous example, the fit indices were not acceptable even for less restricted models. Therefore, the more restricted invariance tests should not be trusted. This is a common problem with CFA. If there is misfit, we can either trim the parameter (drop parameters, variables or groups from the model that are creating problems) or we can add parameters. If we decide to remove items from the model, the overall model needs to be rewritten, with the specific items removed from the revised model (see the steps above). One question that you as a researcher needs to consider is whether removing items may change the meaning of the overall scale (e.g., underrepresentation of the construct; see Fontaine, 2005). It might also be informative for a cross-cultural researcher to consider why a particular item may not work as well in a given cultural context (e.g., through qualitative interviews with respondents or cultural experts to identify possible sources for misfit).

To see which parameters would be useful to add, we can request modification indices.

This can be done using this command in R:

mi <− modificationIndices(fit_cfa)

We could now simply call the output mi to show the modification indices. It gives you the expected drop in *χ^2^* as well as what the parameter estimates would be like if they were freed up. Often, there are many possible modifications that can be done and it is cumbersome sifting through a large output file. It can be useful to print only those modification indices above a certain threshold. For example, if we want to only see changes in χ^2^ above 10, we could add the following argument:

mi <− modificationIndices(fit_cfa, minimum.value = 10,

sort = TRUE)

We also added a command to have the results sorted by size of change in χ^2^ for easier examination. If we now call the object as usual (just write *mi* into your command window), this will give us modification indices for the overall model, that is modification indices for every parameter that was not estimated in the overall model. For example, if there is an item that may show some cross-loadings, we now see how high that possible cross-loading might be and what improvement in fit we would achieve if we were to add that parameter to our model. The function also gives us a bit more information, including the expected parameter change values (column epc) and information about standardized values (sepc.lv: only standardizing the latent variables; sepc.all: standardizing all variables; sepc.nox: standardizing all but exogenous observed variables).

#### Invariance Testing – Individual Restrictions and Partial Invariance

This leads us to the alternative option that we can use for testing invariance. Here, we manually construct increasingly restricted models. This option will also give us opportunities for partial invariance. We first constrain loading parameters in the overall cfa command that we described above:

metric_test <− cfa(cfa_model, data = example,
group = “country”, group.equal = c(“loadings”))

As can be seen here, we added an extra command *group.equal* which now allows us to specify that the loadings are constrained to be equal. If we wanted to constraint the intercepts at the same time, we need to use: group.equal = c(“loadings”, “intercepts”). We can get the usual output using the summary function as described above.

We could now request modification indices for this constrained model to identify which loadings may vary across groups:

mi_metric <− modificationIndices(metric_test,

minimum.value = 10, sort = T)

As before, it is possible to restrict the modification indices that are printed. We could also investigate how much better our model would be if we freed up some parameters to vary across groups. In other words, this would tell us if there are some parameters that vary substantively across groups and if it is theoretically plausible, we could free them up to be group specific. This then would become a partial invariance model (see Meredith, 1993). We provide the *lavTestScore.clean* function in the ccpsyc package to show sample specific modification indices which uses a metrically constrained CFA model. The relevant command is:

lavTestScore.clean(metric.test)

If we wanted to relax some of the parameters (that is running a partial invariance model), we can use the *group.partial* command. Based on the results from the example above, we allowed the third help item to load freely on the help latent factor in each sample:

fit_partial <− cfa(cfa_model, data = example,
group = “country”, group.equal = c(“loadings”),
group.partial = c(“help =∼ help3”))

### Estimating Effect Sizes in Item Bias in CFA: dMACS

The classic approach to multi-group CFA does not allow an estimation of the effect size of item bias. As we did above, when running a CFA to determine equivalence between groups, researchers rely on differences in fit measures such as ΔCFI and Δχ^2^. These cut-off criteria inform researchers whether a structure is equivalent across groups or not, but they do not provide an estimate of the magnitude of misfit. To address this shortcoming Nye and Drasgow (2011) proposed an effect size measure for differences in mean and covariance structures (*d*_MACS_). This measure is estimating the degree of non-equivalence between two groups on an item level. It can be interpreted similar to established effect sizes (Cohen, 1988) with values of greater than 0.20 being considered small, 0.50 are medium, and 0.80 or greater are large. It is important that these values are based on conventions and do not have any direct practical meaning or implication. In some contexts (e.g., high stakes employment testing), even much smaller values might be important and meaningful in order to avoid discrimination against individuals for specific groups. In other contexts, these criteria might be sufficient.

### How to Do the Analysis in R

To ease the implementation of *d*_MACS_, we created a function in R as part of our ccpsyc package that allows easy computation (see the Supplementary Materials for how to install this package and function). The function dMACS in the ccpsyc package has three arguments: *fit.cfa* which takes a lavaan object with two groups and a single factor as input, as well as a *group1* and *group2* argument in which the name of each group has to be specified as string. The function returns effect size estimates of item bias (dMACS) for each item of the factor. In our case, we could specify first a CFA model with only the helping factor, then run the lavaan multi-group analysis.

help_model <− ‘help =∼ help1 + help2 + help3 + help4 + help5 + help6 + help7’
help_cfa <− cfa(help_model, data = example,group = “country”)

We now can call:

dMACS(help_cfa, group1 = “NZ”, group2 = “BRA”)

to get the relevant bias effect size estimates. One of the items (item 3) shows a reasonably large *d*_MACS_ value (0.399). As you will remember, this item also showed problematic loading patterns in the CFA reported above, suggesting that this item might be problematic. Hence, even when the groups may show overall invariance, we may still find item biases in individual items.

### Limitations of dMACS

A limitation of the current implementation of *d*_MACS_ is that the comparison is limited to a unifactorial construct between two groups. After running the overall model, researchers need to respecify their models and test each dimension individually.

### Strengths and Weaknesses of CFA

Confirmatory factor analysis is a theory-driven measurement approach. It is ideal for testing instruments that have a well-established structure and we can identify which items are expected to load on what latent variables. This technique provides an elegant and simple test for all important measurement questions about item properties with multi-dimensional instruments. At the same time, CFA is not without drawbacks. First, it requires interval and multivariate normally distributed data. This can be an issue with the ordinal data produced by Likert-type scales if the data is heavily skewed. Nevertheless a number of studies have shown that potential issues can be overcome by the choice of estimator (for example, Flora and Curran, 2004; Holgado-Tello et al., 2010; Li, 2016). Second, establishing adequate model fit and what counts as adequate are tricky questions and this is continuously debated in the measurement literature. Third, CFA ideally requires moderately large sample sizes (*N* > 200; e.g., Barrett, 2007). Fourth, non-normality and missing data within and across cultural groups can create problems for model estimation and identifying the problems can become quite technical. However, the technique is becoming increasingly popular and has many appealing features for cultural psychologists.

### Exploratory Structural Equation Modeling

Confirmatory factor analysis is a powerful tool, but it has limitations. One of the biggest challenges is that a simple structure in which items only load on one factor is often empirically problematic. EFA (see below) presupposes no structure, therefore any number of cross-loadings are being permitted and estimated, making it a more exploratory technique. To provide a theory-driven test while allowing for the possibility of cross-loadings, ESEM (Asparouhov and Muthén, 2009) has been proposed. ESEM combines an EFA approach that allows an unrestricted estimation of all factor loadings which can then be further compared with a standard structural equation approach. Technically, an EFA is conducted with specific factor rotations and loading constraints. The resulting loading matrix is then transformed into structural equations which can be further tested and invariance indices across groups can be estimated. ESEM also allows a better estimation of the correlated factor-structures than EFA as well as provides more unbiased estimates of factor covariances than CFA (because of the restrictive assumption of a simple structure with no cross-loadings for CFA). The ESEM approach has been proposed within Mplus (Muthén and Muthén, 2018), but it is possible to run compatible models within R (see Guàrdia-Olmos et al., 2013).

We use the approach described by Kaiser (2018). The first step is to run an EFA using the *psych* package.

beh_efa <− fa(example[-1], nfact = 2, rotate = “geominQ”, fm = “ml”)

As before, we are creating an output object (beh_efa) that contains the results of the factor analysis (fa). We specify the data set ‘example’ and the square brackets indicates that we want to run the analysis only for the survey data excluding the country column (example[−1]). We specify 2 factors (nfact = 2) and ask for a specific type of factor rotation that is used by Mplus (rotate = “geominQ”). Finally, we specify a Maximum Likelihood estimator (fm = “ml”).

We now will prepare the output of this EFA to create structural equations that can be further analyzed within a CFA context.

beh_loadmat <− zapsmall(matrix(round(beh_efa$loadings, 2), nrow = 12, ncol = 2))
rownames(beh_loadmat) <− colnames(example[-1])

We use the function *zapsmall* to get the rounded factor loadings from the two factors in the previous EFA (this is the (round(beh_efa$loadings,2) component). The $ sign specifies that we only use the factor loadings from the factor analysis output. We have 12 variables in our analysis, therefore we specify nrow = 12. We have two factors, therefore we specify two columns (ncol = 2). To grab the right variable names, we include a command that assigns the row names in our loading matrix from the respective column (variable) names in our raw data set. Since we have the country variable still in our data set, we need to specify that this column should be omitted: example[−1]. All the remaining column names are taken as the row names for the factor analysis output.

To create the structural equations, we need to create the following loop:


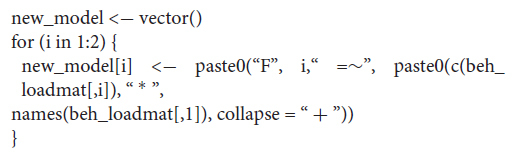


The term i specifies the number of factors to be used. In our case, we have two factors. We then need to specify the relevant loading matrix that we created above (beh_loadmat). If we now call:

new_model

we should see the relevant equations that have been computed based on the EFA and which can be read as a model to be estimated within a CFA approach. Different from our CFA model above, all items are now listed and the loading of each item on the two different factors is now specified in the model.

1 “F1 =∼ 0.55 ^*^ help1 + 0.58 ^*^ help2 + 0.69 ^*^ help3 + 0.91 ^*^ help4 + 0.78 ^*^ help5 + 0.77 ^*^ help6 + 0.51 ^*^ help7 + 0.15 ^*^ voice1 + −0.02 ^*^ voice2 + −0.01 ^*^ voice3 + 0.13 ^*^ voice4 + −0.01 ^*^ voice5”

2 “F2 =∼0.12 ^*^ help1 + 0.1 ^*^ help2 + 0.01 ^*^ help3 + −0.1 ^*^ help4 + 0.04 ^*^ help5 + 0.02 ^*^ help6 + 0.24 ^*^ help7 + 0.65 ^*^ voice1 + 0.85 ^*^ voice2 + 0.77 ^*^ voice3 + 0.65 ^*^ voice4 + 0.8 ^*^ voice5”

We now run a classic CFA, similar to what we did before. We specify that the estimator is Maximum Likelihood (estimator = “ML”). For simplicity, we only want to call some of the fit measures using the fit measures function within lavaan.

beh_cfa_esem <− cfa(new_model, data = example, estimator = “ML”)
fitmeasures(beh_cfa_esem, c(“cfi”, “tli”, “rmsea”, “srmr”))

This analysis was done on the full data set including the Brazilian and NZ data simultaneously, but we are obviously interested in whether the data is equivalent across groups or not (when using this specific model). We can set up a configural invariance test model by specifying the grouping variable and calling the relevant fit indices:

fitmeasures(cfa(

model = new_model,

data = example,

group = “country”,

estimator = “ML”),

c(“cfi”,“tli”,“rmsea”,“srmr”))

If we want to now constrain the factor loadings or intercepts to be equal across groups, we can add the same restrictions as described above. For example, for testing scalar invariance in which constrain both the loadings and intercepts to be equal, we can call this function:

fitmeasures(cfa(

model = new_model,

data = example,

group = “country”,

estimator = “ML”,

group.equal = c(“loadings”, “intercepts”)),

c(“cfi”,“tli”,“rmsea”,“srmr”))

If we compare the results from the ESEM approach with the invariance test reported above, we can see that the fit indices are somewhat better. Above, our CFA model did not show the best fit. Both the CFI and RMSEA showed somewhat less than desirable fit. Using ESEM, we see that the fit of the configural model is better (CFI = 0.947; RMSEA = 0.076) than the original fit (CFI = 0.943, RMSEA = 0.086). Further restrictions to both loadings and intercepts show that the data fits better using the ESEM approach, even when using more restrictive models.

### Limitations

Exploratory structural equation modeling is a relatively novel approach which has been used by some cross-cultural researchers already (e.g., Marsh et al., 2009; Vazsonyi et al., 2015). However, given the relative novelty of the method and small number of studies that have used it, some caution has to be taken. A recent computational simulation (Mai et al., 2018) suggests that ESEM has problems with convergence (e.g., the algorithm does not run), especially if the sample sizes are smaller (less than 200 or the ratio of variables to cases may be too small). Mai et al. (2018) recommended ESEM when there are considerable cross-loadings of items. In cases where cross-loadings are close to zero and the factor structure is clear (high loadings of items on the relevant factors), ESEM may not be necessary. Hence, ESEM might be an appealing method if a researcher has large samples and there are substantive cross-loadings in the model that cannot be ignored.

### Invariance Testing Using Alignment

As yet another extension of CFA approaches, recently Multi-Group Factor Analysis Alignment (from here on: alignment) has been proposed as a new method to test metric and scalar invariance (Asparouhov and Muthén, 2014). This method aims to address issues in MGCFA invariance testing, such as difficulties in establishing exact scalar invariance with many groups. The main difference between MGCFA and alignment is that alignment does not require equality restrictions on factor loadings and intercepts across groups.

Alignment’s base assumption is that the number of non-invariant measurement parameters and the extent of measurement non-invariance between groups can be held to a minimum for each given scale through producing a solution that features many approximately invariant parameters and few parameters with large non-invariances. The ultimate goal is to compare latent factor means, therefore the alignment method estimates factor loadings, item intercepts, factor means, and factor variances. The alignment method proceeds in two steps (Asparouhov and Muthén, 2014). In the first step an unconstrained configural model is fitted across all groups. To allow the estimation of all item loadings in the configural model, the factor means are fixed to 0 and the factor variances fixed to 1. In the second step, the configural model is optimized using a component loss function with the goal to minimize the non-invariance in factor means and factor variances for each group (for a detailed mathematical description see: Asparouhov and Muthén, 2014). This optimization process terminates at a point at which “there are few large non-invariant measurement parameters and many approximately non-invariant parameters rather than many medium-sized non-invariant measurement parameters” (Asparouhov and Muthén, 2014, p. 497). Overall, the alignment process allows for the estimation of reliable means despite the presence of some measurement non-invariance. Asparouhov and Muthén (2014) suggest a threshold of 20% non-invariance as acceptable. The resulting model exhibits the same model fit as the original configural model but is substantially less non-invariant across all parameters considered. Alignment was developed in Mplus (Muthén and Muthén, 2018). Here, we show an example of an alignment analysis using the sirt package (Robitzsch, 2019) which was inspired by Mplus. The exact results may differ between the programs.

### How to Run a Multi-Group Factor Analysis Alignment in R

The *sirt* package provides three useful functions *invariance_alignment_cfa_config*, *invariance.alignment*, and *invariance_alignment_constraints*. These functions build upon each other to provide an easy implementation of the alignment procedure. We use again the example of the helping scale.

We initially fit a configural model across all countries. The *invariance_alignment_cfa_config* makes this straightforward. The function has two main arguments *dat* and *group*; dat takes a data frame as input that only contains the relevant variables in the model. It is important to stress that alignment can currently only fit uni-dimensional models. In our case we select all help variables (help1,…, help7) from the example data set (dat = example[paste0(“help”, 1:7) – the use of the paste0 command selects only the help items from 1 to 7 from the example data set). The group argument takes a grouping variable with the same number of rows as the data provided to the dat argument. In the current case we provide the country column from our data set.

par <− invariance_alignment_cfa_config(dat = example [paste0(“help”, 1:7)], group = example$country)

The *invariance_alignment_cfa_config* function returns a list (in the current case named par) with λ (loadings) and ν (intercepts) for each country and item in addition to sample size in each country and the model fitted. The output of this function can be directly processed in the *invariance.alignment* function. Prior to that the invariance tolerance needs to be defined. Asparouhov and Muthén (2014) suggested 1 for λ and 1 for ν. Robitzsch (2019) utilizes a stricter criterion of λ = 0.40 and ν = 0.20. These tolerances can be varied using the align.scale argument of the *invariance.alignment* function. The first value in a vector provided in this argument represents the tolerance for ν, the second the tolerance for lambda λ. Further, alignment power needs to be set in the align.pow argument. This is routinely defined as 0.25 for λ and ν, respectively. Last, we need to extract λ and ν from the output of the *invariance_alignment_cfa_config* function and provide them to the lambda and nu argument of the *invariance.alignment* function.

mod1 <− invariance.alignment(lambda = par$lambda, nu =

par$nu, align.scale = c(0.2, 0.4), align.pow = c(0.25, 0.25))

The resulting object can be printed to obtain a number of results such as aligned factor loadings in each group and aligned means in each group. We are focusing on the relevant indicators of invariance. *R*^2^ values of 1 indicate a greater degree of invariance, whereas values close to 0 indicate non-invariance (Asparouhov and Muthén, 2014).

mod1$es.invariance[“R2”,]

In our current analysis we obtain an *R*^2^ of 0.998 for loadings and 1 for intercepts. This indicates that essentially all non-invariance is absorbed by group-varying factor means and variances.

Alignment can also be used to assess the percentage of non-invariant λ and ν parameters using the *invariance_alignment_constraints* function. This function takes the output object of the *invariance.alignment* function as input. Additionally, ν and λ tolerances can be specified.

cmod1 <− invariance_alignment_constraints(mod1, lambda_ parm_tol = 0.4, nu_parm_tol = 0.2)
summary(cmod1)

We found that for both factor loadings and factor intercepts none of items exhibited substantial non-invariance (indicated by 0% for the percentage of non-invariant item parameters). Asparouhov and Muthén (2014) suggested a cut-off of 25% non-invariance to consider a scale non-invariant.

### Limitations of Alignment

While alignment is a useful tool for researchers interested in comparisons with many groups, it also has limitations. First, convergence again can be an issue, especially for two group comparisons (Asparouhov and Muthén, 2014). Second, the alignment technique is currently limited to uni-factorial constructs precluding the equivalence test of higher order constructs or more complex theoretical structures. Finally, it is a new method and more work may be necessary to understand practically and theoretically meaningful thresholds and cut-offs in a cross-cultural context.

### Differential Item Functioning Using Ordinal Regression (Item Response Theory)

One of the most common techniques for detecting DIF within the IRT family are logistic regression methods, originally developed for binary response items. It is now possible to use Likert-type scale response options (so-called polytomous items) as ordinal response options. The central principle of DIF testing via logistic regression is to test the probability of answering a specific item based on the overall score of the instrument (as a stand-in for the true trait level, as discussed above). DIF testing via logistic regression assumes that the instrument tested is uni-dimensional. The crucial tests evaluated are whether (a) there are also significant group effects (e.g., does belonging to a specific group make answering an item easier or more difficult, over and above the true trait level) and (b) there are group by ability interactions (e.g., trait effects depend on the group a person belongs to). The first test estimates uniform item bias and the second test estimates non-uniform item bias. Hence, the procedure uses a nested model comparison (similar to CFA invariance testing). A baseline model only includes the intercept. Model 1 includes the estimated true trait level, model 2 adds a dummy for the group (culture) effects and model 3 includes the group (culture) by trait interaction.

We have a number of options to test whether DIF is present. First, it is possible to compare overall model fit using the likelihood ratio chi-square test. Uniform DIF is tested by comparing the difference in log likelihood values between models 1 and 2 (df = 1). Non-uniform DIF is tested by comparing models 2 and 3 (df = 1). It is also possible to test whether there is a total DIF effect by directly comparing model 1 vs. model 3 (df = 2), testing for the presence of both uniform and non-uniform item bias together. This particular approach uses significance tests based on the difference in chi squares.

As we discussed above, chi square tests are sample size dependent, hence a number of alternative tests have been proposed. These alternatives focus on the size of DIF (hence they are effect size estimates of item bias) rather than whether it is significant. There are two broad types: pseudo *R*^2^ (the amount of variance explained by the group effect and group by trait interaction), and raw regression parameters as well as the differences in the regression parameters across models. The interpretation of the pseudo *R*^2^ measures have been debated due to scaling issues (see discussions in Choi et al., 2011), but since we are interested in the differences between nested models, their interpretation is relatively straightforward and similar to normal *R*^2^ difference estimates. Estimates lower than 0.13 can be seen as indicating negligible DIF, between 0.13 and 0.26 showing moderate DIF and above 0.26 large DIF (Zumbo, 1999). As outlined by Choi et al. (2011), some authors have argued that these estimates are too large and lead to under-identification of DIF.

For the regression parameters, it is possible to examine the regression coefficients for the group and the group by trait effects as indicators of the magnitude (Jodoin and Gierl, 2001). It is also possible to examine the difference in the regression coefficient for traits across models 1 and 2 as an indicator of uniform DIF (Crane et al., 2004). If there is a 10% difference in the regression coefficients between model 1 and 2, then this can be seen as a practically meaningful effect (Crane et al., 2004). A convenient feature of the R package that we are describing is that it allows Monte Carlo estimations for detecting DIF thresholds, allowing a computational approach with simulated data for establishing whether items show DIF or not. In other words, the model creates simulated data to estimate how much bias is potentially present in our observed data. The downside is that it is computational demanding and this analysis may take a long time to complete (in our sample using seven items and 2,000 participants, the analysis took over 60 min to complete).

One of the key differences of IRT based approaches compared to CFA is that it refers to differences in item performances between groups of individuals which are matched on the measured trait. This matching criterion is important because it helps to differentiate between differences in item functioning from meaningful differences in trait levels between groups. One of the crucial problems is how to determine the matching criterion if individual items have DIF. The specific package that we describe below uses an iterative purification process in which the matching criterion is recalculated and rescaled using both the items that are not showing DIF as well as group-specific item parameters for items that are found to show DIF. The program is going through repeated cycles in which items are tested and the overall matching score is recalibrated till an optimal solution is found (as specified by the user). This iterative approach is superior to using just the raw scores, but again these iterative processes are computationally more demanding. For more information on the specific steps and computation process, see Choi et al. (2011).

### Logistic Regression to Test for DIF in R

One relevant package that we describe here is lordif (Choi et al., 2011). We chose it because it provides a number of advanced features while being user-friendly. As usual, the package needs to be called as described in the Supplementary Materials. We then need to select only the variables used for the analysis (note the use of the paste0 command again):

response_data <− example[paste0(“help”, 1:7)]

Importantly, the group variable needs to be specified as a vector and is included in a separate file (which needs to be matching to the main data file). In our case, we are using the package car to recode the data:

country <− car::recode(example$country, “’NZ’ = 1; ’BRA’ = 0”)

The actual command for running the DIF analysis is straightforward. In our case, we specify an analysis using the chi-square test:

countryDIF <− lordif (response_data, country, criterion = “Chisqr”, alpha = 0.001, minCell = 5)

As before, we create an output object which contains the results. The function is lordif, which first specifies the data set and then the vector which contains the sample or country information. We then have to make a number of choices. The important choice is to define what threshold we want to set for declaring an item as showing DIF. We can select among χ^2^ differences between the different models (criterion = “Chisqr”, in which case we also need to specify the significance level using the alpha command), *R*^2^ (criterion = “R2”, we need to select the beta.change threshold, e.g., R2.change = 0.01) and the regression coefficients (criterion = “Beta”, we need to select the beta coefficient change, e.g., beta.change = 0.10). These choices can make potentially substantive differences, we urge users to explore their data and decide what criteria is most relevant for their purposes.

A final decision is how to treat minimum cell size (called sparse cell). The analysis proceeds as an ordinal level analysis, if there are few responses to some of the response categories (e.g., very few people ticked 1 or 7 on the Likert scale). We need to specify the minimum value. The default is 5, but we could also specify higher numbers, in which case response categories are collapsed till the minimum cell size is being met by our data. This might mean that instead of having seven response categories, we may end up with five categories only because the extreme response options were combined.

If we use χ^2^ differences as a criterion, item 3 for the helping scale is again flagged as showing item bias. McFadden’s pseudo-R-square values suggest that moving from model 1 to model 2 increases the explained variance by 0.0100, compared to 0.0046 when moving from model 2 to model 3. Hence, uniform item bias is more likely to be the main culprit. The other pseudo-R2 values also show similar patterns. In contrast, if we use the R2 change criterion (and for example, a change of 0.01 as a criterion), none of the items are flagged as showing DIF.

The relevant code is:

countryDIF_r2_change <− lordif(response_data, country,criterion = “R2”, R2.change = 0.01, minCell = 5)

This highlights the importance that selecting thresholds for detecting DIF have for appropriately identifying items that may show problems.

If we wanted to run the Monte Carlo simulation, we write this function (which specifies the analysis to be checked as well as the alpha level and number of samples to be drawn):

countryDIF_MC <− montecarlo(countryDIF, alpha = 0.001, nr = 1000)

### Evaluation of Logistic Regression

There are multiple advantages of using logistic regression approaches within the larger IRT universe. These techniques allow the most comprehensive, yet flexible and robust analysis of item bias. They assume a non-linear relationship between ability and item parameters, which are independent of the specific sample that is being tested. The data needs to be at least ordinal. Both purely statistical significance driven and effect-size based tests of DIF are possible. One distinct advantage is that the lordif package includes an iterative Monte Carlo approach to provide empirically driven thresholds of item bias. Visualization of item bias is also available through the package (see Choi et al., 2011 for details).

At the same time, there are also a number of downsides. First, as with a number of the other techniques mentioned above (*d*_MACS_, alignment), only unidimensional scales can be tested. Second, researchers need to specify thresholds for DIF and the specific choices may lead to quite different outcomes, especially if DIF sizes vary across items. Third, some of the tests are sensitive to sample size and cutoff criteria for DIF differ across the literature. The Monte Carlo simulations are an alternative to construct data-driven cut-offs, but they are computationally intensive. Finally, logistic regression typically requires quite large samples.

### Exploratory Factor Analysis (EFA) and Principal Component Analysis (PCA)

Exploratory factor analysis as a group of statistical techniques is in many ways similar to CFA, but it does not presuppose a theoretical structure. EFA is often used as a first estimation of the factor structure, which can be confirmed in subsequent studies with CFA. Alternatively, researchers may use EFA to understand why CFA did not show good fit. Therefore, EFA is an integral method in the research process and scale development, either as the starting point for exploring empirical structures at the beginning of a research project or for identifying problems with existing scales.

Similar to CFA, the correlations between all items in a test are used to infer the presence of an underlying variable (in factor analytic terms). The two main approaches are proper EFA and Principal component analysis (PCA). The two methods differ conceptually: PCA is a descriptive reduction technique and EFA is a measurement model (e.g., Borsboom, 2006; Tabachnick and Fidell, 2007), but practically they often produce similar results. For both methods, Pearson correlations (or covariances) between observed indicators are used as input, and a component or factor loading matrices of items on components or factors (indicating the strength of relationship of the indicators to the factor in EFA) are the output. For simplicity, we will use the term factor to refer to both components in a PCA and factors in an EFA. More detailed treatment of these methods can be found in other publications (Gorsuch, 1993; Tabachnick and Fidell, 2007; Field et al., 2012).

**FIGURE 4:**
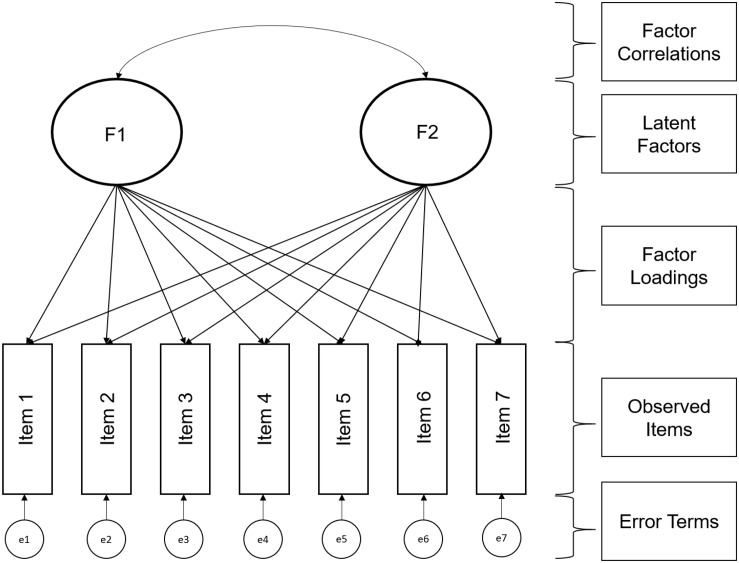
Visual representation of an EFA model.

Figure 4 shows the main parts of an EFA model, which is conceptually similar to the CFA model. One of the major differences is that all items are allowed to load on all factors. As a result, decisions need to be made about the optimal assignment of loadings to factors (a rotational problem, see below) and what constitutes a meaningful loading (an interpretational problem). Items often show cross-loading, in which an item loads highly on multiple factors simultaneously. Cross-loadings of factors may indicate that an item taps more than one construct or factor (item complexity), problems in the data structure, circumplex structures (there is an underlying organization of the latent variables), or it may indicate factor or component overlap (see Tabachnick and Fidell, 2007; Field et al., 2012). As a crude rule of thumb, factor loadings above 0.5 on the primary factor and lack of cross-loadings (the next highest loading varies by at least 0.2) might be good reference points for interpretation.

The principal aim of an EFA is to describe the complex relationship of many indicators with fewer latent factors, but deciding on the number of factors to extract can be tricky. Researchers often use either theoretical considerations and expectations (e.g., the expectation that five factors describe human personality, McCrae and Costa, 1987) or statistical techniques to determine how many factors to extract. Statistical factors take into account how much variance is explained by factors, which is captured by eigenvalues. Eigenvalues represent the variance accounted for by each underlying factor. They are represented by scores that total to the number of items. For example, an instrument with twelve items may capture up to 12 possible underlying factors identified by a single indicator (each item is its own factor). Each factor will have an eigenvalue that indicates the amount of variation that this factor accounts for in the items. The traditional approach to determining the appropriate number of factors was based on Cattell’s scree plot and Kaiser’s criterion that indicates that factors with eigenvalues greater than 1 (e.g., a factor that explains more variance than any item alone is worth extracting). These methods have been criticized for being too lenient (e.g., Barrett, 1986). Statistically more sophisticated techniques such as Horn’s (1965) parallel analysis are now more readily available. Parallel analysis compares the resulting eigenvalues against the eigenvalues obtained from random datasets with the same number of variables and adjusts the obtained eigenvalues (we briefly describe options in the Supplementary Materials).

Once a researcher has decided how many factors to extract, a further important question is how to interpret these factors. First, are the factors assumed to be uncorrelated (orthogonal or independent) or correlated (oblique or related). Latent factor intercorrelations can be estimated when oblique rotation is used (Gorsuch, 1993, pp. 203–204). The choice of rotation is primarily a theoretical decision.

**FIGURE 5:**
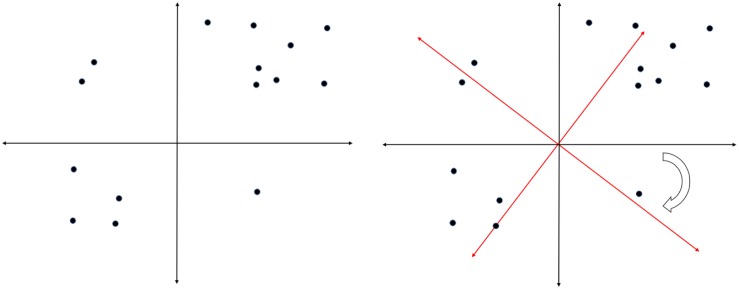
Visualization of factor rotations.

Factor rotations are mathematically equivalent. If more than one component or factor has been identified, an infinite number of solutions exist that are all mathematically identical, accounting for the same amount of common variance. These different solutions can be represented graphically as a rotation of a coordinate system with the dimensions representing the factors and the points representing the loadings of the items on the factors. An example of such rotation is given in Figure 5. Mathematically, the two solutions are identical. Conceptually, we would draw very different conclusions from both versions of the same rotation. This is the core problem with interpreting factor structures across different cultural groups because this rotational freedom can lead to two groups with identical factor structures showing very different factor loadings (see Table 1 for an example – even though the solutions are mathematically identically, they show noticeably different factor loadings). As a consequence, researchers need to rotate their factor structures from the individual groups to similarity before any decisions about factor similarity can be made. The method of choice is orthogonal Procrustes rotation in which the solution from one group is rotated toward the factor structure of the reference group. A good option to decide on the reference group might be to (a) use the group in which the instrument was first developed, (b) use the larger group (since this reduces the risk of random fluctuations that are more likely to occur in smaller groups) or (c) select the group that shows a theoretically clearer or meaningful structure.

**TABLE 1:** An example where identical factor structures show different factor loadings.

	**Factor 1**	**Factor 2**	**Factor 1**	**Factor 2**
Item 1	0.65	0.30	0.67	0.19
Item 2	0.66	0.30	0.69	0.15
Item 3	0.69	0.21	0.80	0.25
Item 4	0.82	0.24	0.80	0.25
Item 5	0.79	0.33	0.67	0.32
Item 6	0.79	0.28	0.71	0.31
Item 7	0.70	0.34	0.39	0.59
Item 8	0.44	0.67	0.22	0.79
Item 9	0.35	0.80	0.19	0.81
Item 10	0.26	0.81	0.23	0.76
Item 11	0.30	0.78	0.43	0.59
Item 12	0.30	0.83	0.23	0.73

After running the Procrustes rotation, the factor structures can be directly compared between the cultural groups. To determine how similar or different the solutions are, we can use a number of different approaches. The most common statistic for comparing factor similarity is Tucker’s coefficient of agreement or Tucker’s phi (van de Vijver and Leung, 1997). This coefficient is not affected by multiplications of the factor loadings (e.g., factor loadings in one group are multiplied by a constant) but is sensitive to additions (e.g., when a constant is added to loadings in one group). The most stringent index is the correlation coefficient (also called identity coefficient). Other coefficients such as linearity, or additivity can be computed, if necessary (for a general review of these options, see van de Vijver and Leung, 1997; Fischer and Fontaine, 2010). Factor congruence coefficients vary between 0 and 1. Conventionally, values larger than 0.85 can be judged as showing fair factor similarity and values larger than 0.95 as showing factor equality (Lorenzo-Seva and ten Berge, 2006), values lower than 0.85 (ten Berge, 1986) are indicative of incongruence. However, these cut-off criteria might vary for different instruments, and no formal statistical test is associated with these indicators (Paunonen, 1997). It is also informative to compare the different indicators, if they diverge from each other this may suggest that there is a problem with the factor similarity.

### Procrustes Rotation With Two Groups Using R

The relevant packages that we need are psych (Revelle, 2018) and GPArotation (Bernaards and Jennrich, 2005). We first need to load these packages and load the relevant data (see the Supplementary Materials for further info).

The first step is to run the factor analysis separately for both samples. We could run either a PCA (using the *principal* function) or factor analysis (using the *fa* function).

nz_fa <− fa(nz_example[,−1], nfactors = 2,
rotate = “varimax”)

We call the factor analysis function (fa**()**) from the psych package and specify the data we are working on (New Zealand data frame without the first column that contains the column with the country information: nz_example [,−1]), the number of factors we want to extract from the data (nfactors = 2), and the rotation we want to use (rotate = “varimax”). Because we have a theoretical expectation we request two factors in each country. We also specify an orthogonal varimax rotation, because we expect the factors to be uncorrelated. Last, we assign the result to an object (nz_fa <−) for later use.

Next, we perform the same action for the Brazilian data using the same procedure:

br_fa <− fa(br_example[,−1], nfactors = 2, rotate = “varimax”)

In the next step, we can directly rotate the factor loading matrices using New Zealand as target matrix and Brazil as loading matrix. In the ccpsyc package, we included a function called prost, which we adapted from the procrustes MCMCpack package in order to provide the identity coefficient and Tucker’s Φ in a straightforward fashion. The *prost* function takes two factor matrices as input and returns the identity coefficient and Tucker’s Φ.

prost(NZ.fa$loadings, BRA.fa$loadings)

We call the output from the factor analyses that we ran above. The $ sign specifies that we only use the factor loadings for the procrustean rotation. In our example, we rotated the Brazilian sample to similarity with the New Zealand sample (first position in our command). We chose NZ as a reference category because NZ is culturally probably more similar to the US where the instrument was developed and the NZ sample was larger, therefore, the solution was expected to be more stable. Tucker’s phi was 0.97 and 0.98, respectively, indicating the factor structures to be equal. The correlation coefficients on the other hand were lower, 0.81 for the first factor (helping) and 0.89 for factor 2 (voice). In addition to the overall factor congruence coefficients, it is also informative to examine the factor structure after rotation to see which items may show higher or lower loadings in each sample.

To do this the prost function has an argument (rotated) which can be set to TRUE (rotated = TRUE). The output now contains the rotated matrix.

prost(NZ.fa$loadings, BRA.fa$loadings, rotated = TRUE)

Researchers can visually compare the differences in factor loadings between the samples to identify any items that may perform differently. These tests are rather subjective and no clear guidelines are available. The interpretation depends on the overall strength of the factor loadings, the number of items and difference in item performance. Unfortunately, no specific statistical tests are available through R that provide more objective tests at the item level. In our example data, one of the voice items showed strong cross-loadings in one sample. Removing this item, the correlation coefficients increased to 0.87 for helping and 0.94 for voice, still not meeting sufficient standards for invariance for at least factor 1 using the more stringent correlation coefficient as a criterion.

### Limitations of the Technique

A major weakness is that the procedure focuses on the congruence at a factorial level, answering whether similar structures are found in each group compared with the reference group. Therefore, we can only establish configural invariance or structural equivalence. Procrustes rotation does not allow to test for metric invariance as the analysis stays at the factor rather than the item level. Individual items may still show substantial loading differences, and the overall factorial similarity might be misleading. For example, research on the structure of the Eysenck Personality Questionnaire has shown that this issue is not without debate (Bijnen et al., 1986; Bijnen and Poortinga, 1988). The number of items and factors may also influence the congruence levels that a researcher can expect to find (Paunonen, 1997). As mentioned above, it is useful to examine the target and target-rotated loadings as well as the difference between the target loadings and the loadings in a norm group to identify potential anomalies in addition to examining any overall congruence coefficient. This may reveal important and useful information of cross-cultural similarities and differences. Nevertheless, Procrustes rotation can be a useful technique at initial research stages. It is also a useful technique if the data does not allow for a full multi-group CFA to be fitted, for example due to a limited number of indicators per construct. Further, Procrustes rotation can be a useful technique to examine the fit of observed structure to an idealized loading matrix of a construct. This process allows a researcher to investigate whether culture level variables significantly impact structural fit.

## Testing Invariance With More Than Two Groups

The most flexible and versatile technique for testing invariance with more than two groups is multi-group CFA. The approach can easily handle more than two groups and no adjustments to the set-up and testing need to be done. One of the challenges is that lavaan provides χ^2^ values for each individual group, but only overall fit indices. Since χ^2^ values are sample size dependent, unless sample sizes are equal, it might be difficult to determine which samples and items are problematic when examining an overall poorly fitting multi-group model. One option is to estimate the individual group models because it will provide important clues about possible problems.

The logistic regression approach implemented in lordif can accommodate more than two groups. However, the visualization of item bias becomes hard to interpret when more than two groups are used.

### Dealing With More Than Two Groups for EFA/PCA

To conduct an EFA/PCA with Procrustes rotation for multiple groups at the same time different methods can be used. Nevertheless, they all require identical steps to set up the data for analysis (we show how to prepare the data for analysis in the Supplementary Materials).

### Target Rotation With Multiple Groups

Conducting an EFA/PCA with a Procrustes rotation to determine configural invariance with more than two samples requires some theoretical considerations:

#### Create an Ideal Matrix as Reference Group

In this option a researcher constructs a loading matrix that represents theoretically assumed loadings on a factor with 1, non-loadings with 0, and negative loadings with −1. This approach is most useful for established measures for which strong theoretical assumptions about the structure exist, such as personality traits. This approach yields insight into the fit of the data from each sample compared to the proposed ideal. Below, we show an example using the *prost* function of the ccpsyc package. We provide an example of how to create an ideal matrix in the Supplementary Materials.

lapply(EFA, function(x){prost(x$loadings, ideal)})

#### Use the Matrix From the Instruments’ Origin

Most questionnaires were and are developed in a Western context. Therefore, a researcher might want to examine how well a newly translated instrument reproduces the structure in regards to the original structure. While this approach can be useful to validate the structure of newly translated instruments in relation to existing data structures, a substantive drawback of this approach is that it posits the origin culture’s structure as de facto correct solution. In our example, we used the results from the first EFA analysis as the target matrix.

lapply(EFA, function(x){prost(x$loadings,EFA[[1]]$loadings)})

#### Creating a Pan-Cultural Matrix

In this approach, an average weighted or unweighted correlation matrix of the items in the structure is created across all cultures of interest. It creates an average matrix, averaging correlations across all items and samples. This does not give priority to any specific cultural group. The resulting correlation matrix can be used as an input to factor analysis and provides a culture-general reference factor loading matrix. This average cultural solution can then be used as the comparison standard for all the individual samples. This approach yields insight into how much each sample corresponds with a common factor solution across all cultures. We show how to create a pan-cultural matrix in the Supplementary Materials. Problems emerge if there is misfit in one or more of the samples and the processes needs to become iterative through pruning mis-fitting samples.

lapply(EFA, function(x){prost(x$loadings,pooled_EFA$loadings)})

#### Choosing a Target Based on Sample Criteria

Sample criteria can also be informative when choosing a rotational target. Considerations such as sample size in each culture and factor simplicity can guide the selection (e.g., the largest sample or the sample with the simplest structure may be selected as comparison). This approach can yield good statistical results but might limit the generalizability of the results and the theoretical interpretation.

#### Running All Pairwise Comparisons

While this approach is free of theoretical considerations, it is only typically feasible or interpretable for a small number of cultures. It is possible to use computational approaches for running cluster analyses of factor similarity, in which we case we attempt to identify groups of samples that show similar factor structures. In the absence of such computational solutions, it might be difficult to make decisions about invariance as one sample might show poor invariance to a second sample, but good invariance to relation to a third sample.

## Overall Comparison of Methods

Which method should you use? There are a number of theoretical questions that can guide you to decide which approach might be best. A first important question is the data that is available. If only ordinal data is available, then IRT remains the most appropriate option. There are options to run CFA and EFA/PCA with ordinal data in R (after computation of polychoric correlations, but these require some intermediate steps). A second important question is whether the researcher has a theoretical model to test or whether the analysis is exploratory. In the former case, both CFA and logistic regression are good options and can be combined to get the most comprehensive insight into the data (Meade and Lautenschlager, 2004a). In the latter case, EFA and PCA are better. New methods such as ESEM are a hybrid that combined EFA with CFA techniques. Third, only CFA-derived methods and logistic regression allow invariance tests at the individual level and statistical tests of DIF. In contrast, EFA and PCA with Procrustes rotation allow only analyses at the scale or instrument level, therefore, they do not provide metric and scalar invariance tests that then would allow the researcher to compare scores directly across groups. Fourth, all techniques described here require decent (ideally *N* > 200) sample sizes, with logistic regression, CFA and associated techniques such as ESEM and alignment being the most sample-size hungry techniques (Meade and Lautenschlager, 2004b). One major drawback for many practical approaches is that logistic regression and alignment (within the CFA-domain) require analyses of unidimensional scales, whereas CFA in particular is versatile in accommodating more complex theoretical structures. Finally, both CFA and logistic regression techniques provide effect size estimates of DIF, which give researchers options to decide how much of bias is too much. Only logistic regression at this moment provides an easily available (but computationally demanding) way to derive empirically derived item bias parameters.

## Summary

Free software for testing invariance at both basic and advanced levels is now available and is easy to use. Comparisons without establishing or testing invariance and equivalence are open to alternative explanations, therefore, invariance testing is paramount. We have highlighted a number of methods and conceptual approaches to allow researchers to test for invariance of their own data. Easy to implement approaches that are free are available to researcher and hopefully will improve the standards and quality of cross-cultural research.

## Author Contributions

RF developed the idea. JK developed the ccpsyc package. RF and JK jointly wrote the manuscript.

## Conflict of Interest Statement

The authors declare that the research was conducted in the absence of any commercial or financial relationships that could be construed as a potential conflict of interest.

## Footnotes

1 https://osf.io/agr5e/

2 Color connotations are often language specific. For example, feeling blue might indicate intoxication in German, but not depression *per se*.

3 The current command ‘measurementInvariance’ available in the semTools package will become unavailable in the near future. A legacy function providing the same output is available in the ccpsyc package via the invariance_semtools function.
