# Supplementary material for: A Primer to (Cross-Cultural) Multi-Group Invariance Testing Possibilities in R
Source: Front Psychol. 2019 Jul 18;10:1507. doi: 10.3389/fpsyg.2019.01507 (PMC6657455; doi:10.3389/fpsyg.2019.01507)
Supplement: Supplementary file 1 [file Table_1.docx]

Supplementary Material:

**A primer to (cross-cultural) multi-group invariance testing possibilities in R**

Ronald Fischer^a,b^ & Johannes Alfons Karl^a^

^a^ Victoria University of Wellington, New Zealand

^b^ Instituto D’Or de Pesquisa e Ensino, Sao Paulo, Brazil

Citation:

Fischer R and Karl J A (2019) A Primer to (Cross-Cultural) Multi-Group Invariance Testing Possibilities in R. Front. Psychol. 10:1507. doi: 10.3389/fpsyg.2019.01507

Supplementary Material:

https://www.frontiersin.org/articles/10.3389/fpsyg.2019.01507/full#supplementary-material

Introduction to R

# A short introduction to R

The R language is widely used among statisticians, data miners, and researchers for data analysis. R’s popularity has increased substantially in recent years, ranking 8th in the TIOBE index.

In this section we will provide a brief introduction and starter guide to R, which should help readers to understand how R works and how to load data for analyses. There are many useful guides and introductions, for example a more thorough introduction can be found in the [CRAN project repository](https://cran.r-project.org/doc/manuals/r-release/R-intro.pdf) and there are many resources at various level available on github (for example, <https://github.com/hadley/adv-r>; ). R is a free programming language and software environment for statistical computing (for download links see here: [Windows](https://cran.r-project.org/bin/windows/base/), [Mac](https://cran.r-project.org/bin/macosx/)). New versions of R are released periodically and can be downloaded and installed to replace the older R version. While the base R command line interface is sufficient for analysis purpose, we recommend installing an Integrated Development Environment (IDE) such as [R Studio](https://www.rstudio.com/products/rstudio/download/). R Studio provides an interface with a number of user-friendly options, including a separate console and editor that has various help and syntax-autocomplete functions, and various tools for plotting, history, data visualization, debugging and workspace management.

#

# The basic structure of R commands

The four main elements of every R code are objects, functions, arguments, and operators. R is an object-based language and Figure 1 shows a simplified representation of these elements.
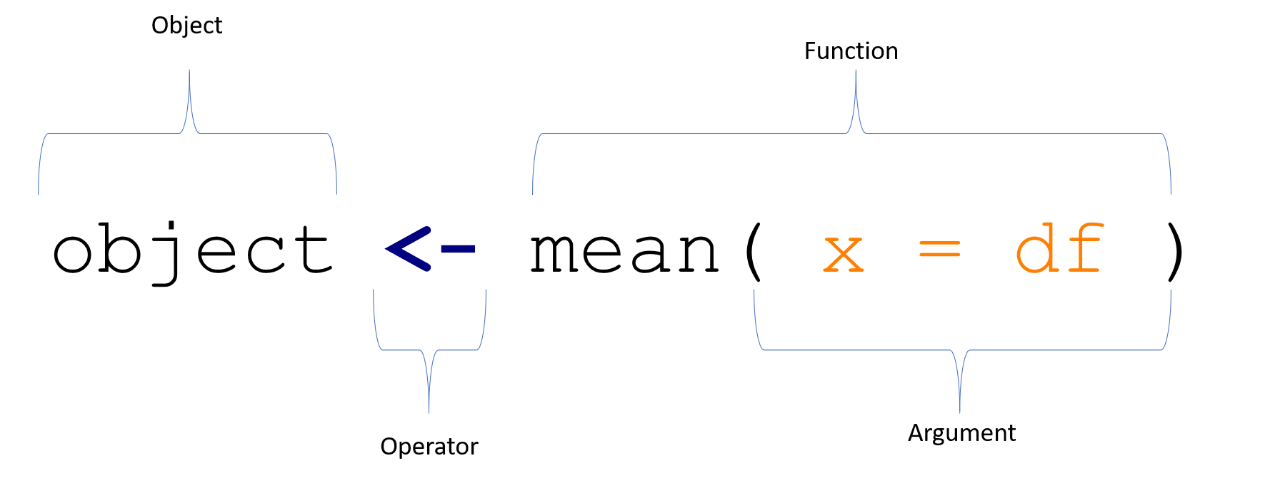


Figure 1 provides a simple example, that produces a new object which contains the mean of variable x.

The function **mean** generates the arithmetic mean of an input object. The object needs to be specified inside of the function brackets as the **x** argument, in this case we define **x = df** . Last, we assign the result of this function for later us via the **<-** operator to an object which we decided to call **object**.

In other words, we create a new object that can be further manipulated and contains information about the mean of a previously created object ‘x’. This structure represents the foundation of most operations in R.

Instead calling the mean function as above one could manually add all values of x dividing it by the number of x values. Nevertheless, this would be very cumbersome. For this reason, functions, pre-assembled lines of code, exist to reduce the amount of coding necessary. These functions can be bundled into packages, which is the main appeal of R as a statistical tool because community developed functions are available from a central repository called CRAN in the form of packages. These packages can be installed in R with the command *install.packages(“package name”)*.

For some of the functions described in the main text, we developed a proto-package. It can be installed running this function (requires installation of the devtools package first:

install.packages(“devtools”)

devtools::install_github("Jo-Karl/ccpsyc")

After you have downloaded the packages that you need for your analysis, the packages need to be loaded into the current R working environment using *library(“package name”).* For example, if we want to use the lavaan package, we call: library(lavaan)

To find relevant packages, a good starting point is to visit R specific search engine sites such as [rseek](http://rseek.org/) (https://rseek.org).

Before describing the specific applications of packages and commands for running equivalence and invariance tests, we briefly describe how to get started and load data into the R environment. This is primarily for readers unfamiliar with this environment, more experienced users of R can skip this section.

**Projects in R Studio**

We recommend that users starting out with R use R studio projects to organize their workflow and files. Projects can be generated by opening the *file* tab in the left corner of R studio and selecting New Project. Users can then either select to create a new directory (new folder) or use an existing directory (existing folder). We recommend for this tutorial to create a new directory by selecting this option and in the subsequent window to select a new project as project type. This directory can be named and placed at the user’s convenience. All data and code should be stored within this newly created directory, which simplifies processing and loading data and objects. If a user wants to start an R work session for this specific project it should be launched via the .rproj file in the new directory. For a more detailed description with screen shots for easy reference we recommend *R for data Science* which is freely available: https://r4ds.had.co.nz/workflow-projects.html

# Importing data into the R environment

We first want to show how data can be included in the R environment for later analysis. We focus on .csv data, but the procedure is similar for [.xslx](https://cran.r-project.org/web/packages/xlsx/xlsx.pdf), [.spss, .sas, .stata](https://cran.r-project.org/web/packages/foreign/index.html). We are using R Studio projects to avoid manually setting working directories, which would otherwise be necessary. R Studio provides a succinct introduction how to create projects using [R Studio](https://support.rstudio.com/hc/en-us/articles/200526207-Using-Projects).

To read .csv data into the R environment we use the read.csv function. Below is an example for the current data set:

example <- read.csv("data/example.csv", na.strings = "999")

We read a data set called example.csv located in the data folder we created in our R project. The quotation marks indicate that "data/example.csv" should be read as text-string, rather than referring to a previously defined object. The additional argument na.strings = "999" specifies that all values in all columns that are 999 should be treated as missing values. The choice of the value 999 is arbitrary but often used by researchers to indicate missing values in data sets. In case that a researcher has used different missing values, this argument needs to be adapted. If there are multiple missing value options or different values set for different variables, we would recommend other options (for good examples, see Field, Miles, Field, 2012). It is important to note that different functions within R may not be operating properly when some data are missing. This needs to be carefully checked by the researcher. Finally, example <- indicates that we want to save the data set as object called example. This will create a new data object which contains the .csv data in a format that is readable in R and allows us to do further work with it.

The current data set had a number of missing observations. We declared all missing values as NA, which is R’s indicator for ‘not available’, and this allows us to later include or exclude such cases when running further analyses. Depending on the research question and the number of missing cases different treatments such as replacing missing values might be appropriate (Little, Rubin, 2002). R provides an easy way to drop all cases with missing observations with the na.omit function. It is possible to omit all cases with missing data in a listwise fashion. For example, we could overwrite the current object (data frame) by applying the function to the data set *example* we defined in the previous step and assign it again to an object called *example*.

example <- na.omit(example)

If we wanted to create a new data frame, we could give the new object a different name.

example.nomissing <- na.omit(example)

Data frame manipulation is an essential skill for the effective usage of R. For our examples we first need to select our variables of interest from a larger data frame. For this, we use the select function from the dplyr package (Wickham, François, Henry, & Müller, 2018). Remember to first download and load the dplyr package.

example.small <- select(example, c("country", "help1", "help2", "help3", "help4", "help5", "help6", "help7", "voice1", "voice2", "voice3", "voice4", "voice5"))

Throughout our examples using two-group comparisons, we use the data from Brazil and New Zealand. This sub-set of our data can be selected by filtering the full dataset to only contain data from New Zealand and Brazil. We are using the filter function from the dplyr package (remember to install and load this package first).

example <-filter(example.small, country %in% c("BRA", "NZ"))

If only a single country is used, for example to conduct an EFA, we again use the filter function to select samples from individual countries .

data.nz <- filter(example.small, country == "NZ")

**Setting up data and running a multi-group EFA**

First, we need to split our complete dataset with 8 countries into 8 data frames. This can be achieved with a single command line.

split_list<- split(overall, overall$country)

The split command from base R takes as first argument a data frame (overall) and as second argument a variable upon which to split (overall$country). The resulting object split_listis a list that contains an individual data frame for each country.

In the next step we want to remove the first column from each data frame in the list, as it now contains a constant for each country.

variable_list <- lapply(split_list, function(x){x[,-1]})

The command lapply takes a list (split_list) as input and applies a function (function(x){x[,-1]}) to each element that is part of it. In this case we defined a function that removes the first column (as indicated by [,-1]), which in our case is the country variable, from each data frame.

Finally, we want to run the same EFA for each country.

EFA <- lapply(variable_list, function(x){fa(x, rotate = "varimax", nfactors = 2, use=”pairwise”)})

To do this we again use lapply to apply the factor analysis function from the psych package to the list of data frames. The resulting object (EFA) contains the output of each EFA for each country.

**Creating an ideal matrix for a multi group EFA**

In our example we expect a bi-factorial solution with each of the items only loading on one factor. To create the idealised matrix, we can use the following code:

ideal <- matrix(c(1,0,1,0,1,0,1,0,1,0,1,0,1,0,0,1,0,1,0,1,0,1,0,1), byrow = TRUE, ncol = 2)

We specify the positive and null loadings matrix(c(1,0,1,0,1,0,1,0,1,0,1,0,1,0,0,1,0,1,0,1,0,1,0,1)) for each item on each factor. Next, we specify that the resulting matrix should be filled by row and that we want a matrix with two columns to be created. Below is the resulting matrix object:

| 1 | 0 |
| --- | --- |
| 1 | 0 |
| 1 | 0 |
| 1 | 0 |
| 1 | 0 |
| 1 | 0 |
| 1 | 0 |
| 0 | 1 |
| 0 | 1 |
| 0 | 1 |
| 0 | 1 |
| 0 | 1 |

The next step is to rotate the factor solution obtained in each country to the ideal matrix and examine the congruence coefficients. This can be done using the command from the main text:

EFA <- lapply(variable_list, function(x){efa(x, rotate = "varimax", nfactors = 2, use=”pairwise’”)})

lapply(EFA, function(x){ prost(x$loadings, ideal)})

**Creating a pan-cultural matrix**

To create a pan-cultural correlation matrix that weights each sample equally when creating the average correlation matrix (each country has the same influence on the final result independent of the sample size). We use the statsBy function from the psych package. Importantly, we need to first convert the grouping variable into a numeric variable.

overall$country_numeric <- as.numeric(factor(overall$country))

The above command drops the character factor levels leaving us with the numbers assigned for each country.

pooled_correlation <- psych::statsBy(data = overall[-1], group = "country_numeric",

cors = TRUE, cor = "cor", method ="pearson")

pooled_efa <- psych::fa(r = pooled_correlation$pooled, nfactors = 2,

rotate = "varimax")

The statsBy function takes as first argument (data) a data frame with the variables that are needed to create the correlation matrix and a grouping variable, such as country. We specify that we want the *overall* dataset but drop the first column (overall-1]) as we do not want to include the country column in the summary. The second argument (group) specifies the variable name of the grouping variable. The function then specifies the relevant correlation parameters (using Pearson correlation). We then run the factor analysis on the pooled correlation matrix. The subsequent steps are identical to the other methods presented in the main text and can be called:

output3_efa <- lapply(variable_list, function(x){fa(x, rotate = "varimax", nfactors = 2)})

lapply(output3_efa, function(x){prost(x$loadings, pooled_efa$loadings)})

If we have many samples, it might be useful to create a more reader friendly table. We first create an object that contains the output of our last analysis and then convert the list created by lapply back into a data frame and include the country code values to help us with the interpretation of the indices.

procrustes_output <- lapply(output3_efa, function(x){prost(x$loadings, pooled_efa$loadings)})

result_table <-as.data.frame(cbind(names(procrustes_output), Reduce("rbind", lapply(procrustes_output,unlist))))
